# Ferric carboxymaltose in patients with pulmonary arterial hypertension and iron deficiency: a long‐term study

**DOI:** 10.1002/jcsm.12764

**Published:** 2021-09-09

**Authors:** Tilmann Kramer, Max Wissmüller, Kristiana Natsina, Felix Gerhardt, Henrik ten Freyhaus, Daniel Dumitrescu, Thomas Viethen, Martin Hellmich, Stephan Baldus, Stephan Rosenkranz

**Affiliations:** ^1^ Klinik III für Innere Medizin Herzzentrum der Universität zu Köln Cologne Germany; ^2^ Cologne Cardiovascular Research Center (CCRC) Heart Center at the University of Cologne Cologne Germany; ^3^ Klinik für Allgemeine und Interventionelle Kardiologie Herz‐ und Diabeteszentrum NRW Bad Oeynhausen Germany; ^4^ Institut für Medizinische Statistik, Informatik und Epidemiologie (IMSIE) Universität zu Köln Cologne Germany

**Keywords:** Pulmonary arterial hypertension (PAH), Iron deficiency, Iron, Ferric carboxymaltose

## Abstract

**Background:**

Pulmonary arterial hypertension (PAH) is a progressive disease with limited survival. Iron deficiency (ID) correlates with disease severity and mortality. While oral iron supplementation was shown to be insufficient in such patients, the potential impact of parenteral iron on clinical measures warrants further investigation.

**Methods:**

We retrospectively analysed the long‐term effects of intravenous ferric carboxymaltose (FCM) on iron status and clinical measures in patients with PAH and ID [ferritin < 100 μg/L or ferritin 100–300 μg/L and transferrin saturation (TSAT) < 20%] who were on stable targeted PAH therapy, compared with matched controls without ID. Patients with ID received a single infusion of FCM (500 to 1000 mg). Clinical measures monitored included exercise capacity, World Health Organization (WHO) functional class, ESC/ERS risk status, and hospitalizations. The observation period was up to 18 months.

**Results:**

One hundred and seventeen patients (mean age 60.9 ± 16.1 years; 64.1% females) with confirmed PAH and on stable targeted therapy for ≥3 months were included (58 with and 59 patients without ID who did not receive FCM). In patients with ID, iron supplementation with FCM resulted in an immediate and sustained improvement of iron status for up to 18 months (serum iron, ferritin, TSAT, all *P* < 0.01). Fourteen patients in the FCM group received a second FCM infusion after 9.6 ± 4.8 months due to recurrent ID. At 6 and 18 months after FCM infusion, 6 min walk distance improved from 377.5 ± 15.9 at baseline to 412.5 ± 15.1 and 400.8 ± 14.5 m, respectively (both *P* < 0.05). WHO functional class (*P* < 0.05) and ESC/ERS risk status also improved, and there was a reduction of hospitalizations for worsening PAH in the 12 months post vs. prior to iron repletion (*P* = 0.029). No significant changes were observed in the control group. FCM was well tolerated in all patients, with no severe adverse events.

**Conclusions:**

In addition to targeted therapy, correction of ID by parenteral iron supplementation with FCM appears feasible and safe, has sustained effects on iron status, and may improve the clinical status and hospitalization rates in patients with PAH. Larger controlled studies are required to confirm this finding.

## Introduction

Pulmonary arterial hypertension (PAH) remains a limiting and progressive disease, defined by a mean pulmonary arterial pressure (PAP) ≥ 25 mmHg and normal pulmonary arterial wedge pressure (PAWP ≤ 15 mmHg) at rest as assessed by right heart catheterization (RHC).^1^, ^2^ Recent morbidity/mortality‐driven randomized controlled trials showed that initial or sequential combination therapy with PAH‐targeted drugs substantially improve outcome, but clinical endpoints occurred in approximately one third of patients even under optimized conditions of combination therapy.^3^, ^4^, ^5^ Besides targeted treatment, systemic consequences of PAH and right‐sided heart failure—including iron deficiency (ID)—may represent additional treatable traits.^6^ In this context, current guidelines also recommend supportive therapy such as diuretics, supervised exercise training, supplemental oxygen, and possibly the correction of ID and anaemia.^1^, ^7^

Several studies indicated that ID is common in PAH patients, occurring with a prevalence of 43–60% in idiopathic PAH (IPAH) or heritable PAH, and those with PAH associated with systemic sclerosis or Eisenmenger's syndrome.^7^, ^8^, ^9^ ID was associated with reduced exercise capacity, higher symptom burden and mortality in all mentioned PAH subgroups, independent of the presence of anaemia.^7^, ^8^, ^9^, ^10^ ID may contribute to symptom burden and morbidity via multiple mechanisms including impairment of heart, inspiratory, and skeletal muscle.^11^, ^12^, ^13^ A potential explanation for the presence of ID in PAH patients may be a reduced dietary iron uptake due to elevated concentrations of hepcidin, an important regulating protein of iron metabolism that plays a central role in inhibiting enteral iron uptake.^10^, ^14^

While oral iron supplementation appeared insufficient in PAH‐associated ID,^7^, ^8^, ^10^ in a previous open‐label pilot study in 20 PAH patients with ID, we found that a single infusion of ferric carboxymaltose (FCM) led to short‐term improvements of the 6 min walk distance (6MWD) and quality of life.^15^ Consistently, a further study performed in 15 patients with PAH also found beneficial effects of intravenous (i.v.) iron supplementation on exercise capacity, time to anaerobic threshold, and improved oxygen handling through increased myoglobin and mitochondrial oxidative capacities.^16^ These data are in line with clinical improvements upon i.v. iron therapy in patients with chronic left‐sided heart failure (HF).^17^, ^18^, ^19^, ^20^

Although current pulmonary hypertension (PH) guidelines and consensus recommendations indicate that correction of ID in PAH patients may be considered,^1^, ^7^ solid data on the long‐term effects in a sufficient number of patients are lacking, and the sustainability of i.v. FCM on iron status (i.e. the necessity for repetitive iron supplementation) in PAH remains unknown. Therefore, we investigated the feasibility and potential long‐term effects of i.v. iron supplementation with FCM in an extended cohort of patients with PAH and ID over a period of 18 months. In addition to assessing iron status and standard measures of exercise tolerance and symptom burden, we also monitored the impact on PAH risk status (ESC/ERS) and the rate of PAH‐associated hospitalizations.

## Patients and methods

### Study population

We retrospectively analysed 117 patients with PAH (Nice group 1; between December 2010 and October 2018), 58 of whom had ID and received i.v. FCM (intervention group), whereas 59 subjects with PAH of similar origin without ID, who did not receive FCM, served as ‘matched’ controls (control group). In all patients, PAH was confirmed by RHC as recommended by current guidelines.^1^ Patients were selected to approximately frequency‐match for gender, age, type of PAH, type and duration of treatment, comorbidities, and haemodynamics. Patients with kidney dysfunction (serum creatinine > 2.0 mg/dL), considerable liver disease (serum glutamic oxaloacetic transaminase/glutamic pyruvic transaminase > 70 U/L), marked anaemia (haemoglobin < 8.0 mg/dL) or inflammation [C‐reactive protein (CRP) > 25 mg/L], and those not on stable targeted PAH therapy were excluded (*Figure* S1). FCM was administered within the approved label (ID in (right) HF). Both groups were monitored for 18 months, during which follow‐up visits were performed at 3, 6, 12, and 18 months after the first FCM administration, assessing 6MWD, laboratory measures, World Health Organization functional class (WHO‐FC), and echocardiography. All patients gave written informed consent for the usage of their data for research purposes, and the analysis was approved by the local Ethics Committee of the University of Cologne (reference 20‐1647).

### Definition of iron deficiency

Iron status was assessed by measuring serum ferritin (normal values females: 30–150 μg/L; males: 30–400 μg/L), iron (9.0–30.0 μmol/L), and transferrin saturation (TSAT) (16–45%) by standard laboratory procedures (accredited by ISO 15189). The concentrations of haemoglobin (normal values: females 12.0–16.0 g/dL; males: 13.5–18.0 g/dL), mean corpuscular volume (MCV; 80–96 fL), creatinine, NTproBNP, and CRP were also determined. ID was defined by a serum ferritin < 100 μg/L or a serum ferritin of 100 to 300 μg/L in combination with a TSAT < 20%, in the absence of significant inflammation (CRP < 25 mg/L),^17^, ^18^, ^20^ consistent with current HF guidelines.^21^

### Right heart catheterization

Pulmonary arterial hypertension was confirmed by RHC in all patients and was defined by a mean (PAP) ≥ 25 mmHg, PAWP ≤ 15 mmHg, and pulmonary vascular resistance > 3 Wood units at rest.^1^, ^2^ All pressure values were measured at end‐expiration, and as per standardized RHC protocol, the pressure transducer was routinely placed at the mid‐thoracic level. RHC included the measurement of cardiac output, mixed venous oxygen saturation, and calculation of transpulmonary pressure gradient (mean PAP‐PAWP), diastolic pressure gradient (diastolic PAP‐PAWP), and pulmonary vascular resistance [(mean PAP‐PAWP)/cardiac output]. Significant left heart or lung disease was routinely ruled out, and chronic thromboembolic PH was excluded by ventilation/perfusion scan, according to current guidelines.^1^, ^2^

### Transthoracic echocardiography

Transthoracic echocardiography was performed using the Philips iE 33 system (Philips GmbH, Hamburg, Germany), equipped with a 2.5 MHz transducer. Assessments of right heart morphology and function included right atrial area, right ventricular end‐diastolic diameter, tricuspid annular plane systolic excursion, and tricuspid regurgitation velocity. The systolic tricuspid pressure gradient (∆*P*_max_TV) was calculated from tricuspid regurgitation velocity by the modified Bernoulli equation, and pulmonary arterial systolic pressure was estimated as the sum of ∆*P*_max_TV and right atrial pressure. Additionally, left ventricular ejection fraction was evaluated. All measurements were performed according to current guidelines.^1^

### Six‐minute walk test

Exercise capacity was evaluated by the 6MWD. The 6 min walk test, in which the patient walks as far as possible within 6 min time, was performed in a standardized way according to the guidelines of the American Thoracic Society.^22^ The estimated consensus minimal clinical important difference in the 6MWD for PAH is approximately 33 m.^23^ All subjects were familiar with the test and were used to perform it on a regular basis at their routine follow‐up visits.

### Intervention

Iron‐deficient PAH patients received i.v. FCM [Ferinject®; Vifor Inc., St. Gallen, Switzerland (FCM)]. The FCM complex is a dextran‐free iron formulation consisting of a polynuclear iron (III) hydroxide complexed to carboxymaltose, which can be administered in high doses without releasing large amounts of reactive iron into the circulation or triggering dextran‐associated immunogenic reactions.^24^ In our study, FCM was given up to a maximum single dose of 1000 mg but not exceeding 15 mg of iron per kilogram body weight, infused over a 15 min period. Iron status was assessed periodically, and repetitive FCM infusions were administered in cases of recurrent ID. Every FCM infusion was given after completion of all clinical assessments. Adverse events were recorded on a regular basis. No patient in the control group received FCM at any time during the observation period.

### Risk assessment

Risk assessment for PAH was performed according to current guidelines,^1^ using (i) the SPAHR/COMPERA approach and (ii) the French non‐invasive three‐criteria approach including WHO‐FC, 6MWD, and NTproBNP.^25^, ^26^, ^27^ (i) Overall risk assessment (‘low’, ‘intermediate’, and ‘high’ risk status): patients were categorized as ‘Low risk’, ‘Intermediate risk’, or ‘High risk’ according to cut‐off values for WHO‐FC, 6MWD, NTproBNP, right atrial area, mean right atrial pressure, pericardial effusion, cardiac index, and mixed venous oxygen saturation defined in the ESC/ERS guidelines.^1^ Each variable was graded from 1 to 3 where 1 = ‘Low risk’, 2 = ‘Intermediate risk’, and 3 = ‘High risk’. If a 6 min walk test was registered as interrupted, it was assigned a grade of 3. Dividing the sum of all grades by the number of available variables for each patient rendered a mean grade. The mean grade was rounded off to the nearest integer, which was used to define the patient's risk group.^27^, ^28^ (ii) Identification of ‘low‐risk status’ using the non‐invasive three‐criteria approach by counting the number of low‐risk criteria: low‐risk criteria were defined as NYHA/WHO‐FC I or II, 6MWD > 440 m, and NTproBNP < 300 ng/L.^1^, ^25^, ^26^ Patients were classified according to the number of low‐risk criteria present at the various time points.

### Hospitalizations

The hospitalization rate of the intervention group was recorded for a period of 12 months after first administration of FCM, compared with the 12 month period prior to FCM treatment, presumably under conditions of ID. Hospitalization rates of the control group were recorded for the corresponding time frames. PAH‐associated hospitalizations were defined as hospitalizations due to signs of decompensated right HF associated with dyspnoea and/or pericardial effusion and/or peripheral oedema and/or ascites and/or requiring i.v. diuretic therapy and/or escalation of PAH targeted therapy. According to these criteria, hospitalizations were independently adjudicated by three experienced clinicians with expertise in PH (TK, FG, and SR).

### Statistical analysis

Qualitative variables were summarized by count and percentage, quantitative variables by mean ± standard deviation, or median (25th to 75th percentile, refer to the supporting information). Changes in mean values of quantitative variables (i.e. before and after treatment) were described by mean ± standard error and evaluated using the paired *t*‐test at two‐sided significance level 5%. Differences in distributions between groups were evaluated by the Mann–Whitney *U*‐test (quantitative data) or Fisher's exact test (qualitative data). Paired time‐to‐event distributions were summarized by the Kaplan–Meier method and compared by the Wilcoxon signed‐rank test. *P* values < 0.05 were considered statistically significant. Last Observation Carried Forward imputation was used for missing values. Calculations were done with Excel (Microsoft Corp., Redmond, WA, USA) and SPSS Statistics (IBM Corp., Armonk, NY, USA).

## Results

### Patient characteristics

Demographics and patient characteristics of the 117 patients are presented in *Table* 1. At baseline, the intervention and control groups were well‐matched, with no significant differences in age, gender, aetiology of PAH, and comorbidities. In both groups, IPAH was the primary type of PAH. Patients were on a stable PAH therapy with a phosphodiesterase type 5 inhibitor or soluble guanylate cyclase stimulator, endothelin receptor antagonist, or prostanoid/prostacyclin receptor agonist (oral/inhaled) for ≥3 months prior to first FCM administration (≙ baseline). Two patients (one in the intervention and one in the control group) had a positive ‘vasoresponder status’ and were therefore treated with a calcium channel blocker (amlodipine). Concomitant medication is listed in Supporting Information, *Table* S1. In the intervention group, PAH therapy was escalated in 15 patients (25.9%) during the 18 month follow‐up, compared with 21 patients (35.6%) in the control group. The average time to first escalation in the intervention group was 4.5 ± 2.7 months vs. 8.9 ± 3.5 months in the control group.

**Table 1:** Demographics and patient characteristics, at baseline in the intervention group (FCM) and in the control group (no FCM)

Variable	Intervention (*n* = 58)	Control (*n* = 59)	*P* value
Patient characteristics			
Mean age (years)	58.3 ± 18.4	63.4 ± 13.0	0.253
Median age, years (IQR)	60.5 (44.8, 74.8)	65.0 (55.0, 73.5)	0.254
Gender, f/m (%)	70.7/29.3	57.6/42.4	0.178
Weight (kg)	72.0 ± 16.8	81.2 ± 17.7	0.006
Height (cm)	167.0 ± 9.5	168.8 ± 8.5	0.211
Aetiology of PAH			
Idiopathic, *n* (%)	37 (63.8%)	32 (54.2%)	0.349
Hereditary, *n* (%)	6 (10.3%)	2 (3.4%)	0.163
Drug‐induced, *n* (%)	0 (0.0%)	0 (0.0%)	—
CTD, *n* (%)	10 (17.2%)	19 (32.2%)	0.086
CHD, *n* (%)	2 (3.4%)	1 (1.7%)	0.619
HIV, *n* (%)	1 (1.7%)	0 (0.0%)	0.496
Other, *n* (%)	2 (3.4%)	5 (8.5%)	0.439
Comorbidities/CV risk factors			
Hypertension, *n* (%)	28 (48.3%)	36 (61.0%)	0.196
Diabetes, *n* (%)	7 (12.1%)	13 (22.0%)	0.219
Dyslipidaemia, *n* (%)	15 (25.9%)	22 (37.3%)	0.234
CAD, *n* (%)	13 (22.4%)	16 (27.1%)	0.669
BMI > 30 kg/m^2^, *n* (%)	8 (13.8%)	15 (25.4%)	0.162
Targeted PAH therapy at baseline			
CCB mono, *n* (%)	1 (1.7%)	1 (1.7%)	≈1.000
PDE5i mono, *n* (%)	13 (22.4%)	21 (35.6%)	0.209
ERA mono, *n* (%)	5 (8.6%)	5 (8.5%)	≈1.000
sGC‐S mono, *n* (%)	5 (8.6%)	3 (5.1%)	0.490
PDE5i + ERA, *n* (%)	22 (37.9%)	25 (42.4%)	0.707
sGC‐S + ERA, *n* (%)	3 (5.2%)	1 (1.7%)	0.364
PDE5i + ERA + PCA, *n* (%)	6 (10.3%)	2 (3.4%)	0.163
sGC‐S + ERA + PCA, *n* (%)	3 (5.2%)	1 (1.7%)	0.364

CAD, coronary artery disease; CCB, calcium channel blocker; CHD, congenital heart disease; CTD, connective tissue disease; ERA, endothelin receptor antagonist; PCA, prostacyclin analogue; PDE5i, phosphodiesterase‐5 inhibitors; sGC‐S, soluble guanylate cyclase.

The most common type of PAH therapy was a combination of phosphodiesterase type 5 inhibitor and endothelin receptor antagonist in both groups (*Table* 1). Moreover, the use of anticoagulants was similar in both groups. Cardiopulmonary haemodynamics are summarized in *Table* 2, demonstrating pre‐capillary PH and a comparable haemodynamic pattern in both groups.

**Table 2:** Cardiopulmonary haemodynamics as assessed by right heart catheterization in the intervention and control group

Baseline	All (*n* = 117)	Intervention (*n* = 58)	Control (*n* = 59)	*P* value
Systolic PAP (mmHg)	68.3 ± 23.3	69.1 ± 23.9	67.3 ± 22.7	0.817
Diastolic PAP (mmHg)	25.3 ± 11.0	26.2 ± 11.8	24.3 ± 9.9	0.575
Mean PAP (mmHg)	42.4 ± 13.8	43.2 ± 13.8	41.7 ± 13.7	0.749
PAWP (mmHg)	11.9 ± 5.5	11.8 ± 4.4	12.0 ± 6.6	≈1.000
RAP (mmHg)	8.6 ± 4.2	8.3 ± 4.6	9.0 ± 3.8	0.244
CO (L/min)	4.6 ± 1.1	4.5 ± 1.2	4.7 ± 1.1	0.481
CI (L/min/m^2^)	2.5 ± 0.5	2.5 ± 0.5	2.4 ± 0.5	0.799
TPG (mmHg)	31.0 ± 14.4	32.0 ± 14.7	29.8 ± 14.1	0.566
DPG (mmHg)	14.3 ± 11.2	14.7 ± 12.1	14.2 ± 10.0	0.905
Heart rate (bpm)	74.1 ± 12.6	74.1 ± 14.3	74.1 ± 10.8	0.695
SVI (mL/m^2^ per beat)	34.4 ± 9.2	36.0 ± 10.1	33.0 ± 8.1	0.066
PVR (WU)	8.0 ± 4.6	8.1 ± 4.9	7.9 ± 4.2	0.861
PAC (mL/mmHg)	1.7 ± 0.8	1.7 ± 0.8	1.7 ± 0.9	0.799
SvO_2_ (%)	65.9 ± 7.6	64.2 ± 8.0	67.7 ± 6.7	0.040

CI, cardiac index; CO, cardiac output; DPG, diastolic pressure gradient; PAC, pulmonary arterial capacitance; PAP, pulmonary arterial pressure; PAWP, pulmonary arterial wedge pressure; PVR, pulmonary vascular resistance; RAP, right atrial pressure; SVI, stroke volume index; SvO_2_, mixed venous oxygen saturation; TPG, transpulmonary pressure gradient; WU, Wood units.

### Iron supplementation with ferric carboxymaltose replenished iron status

**Figure 1:**
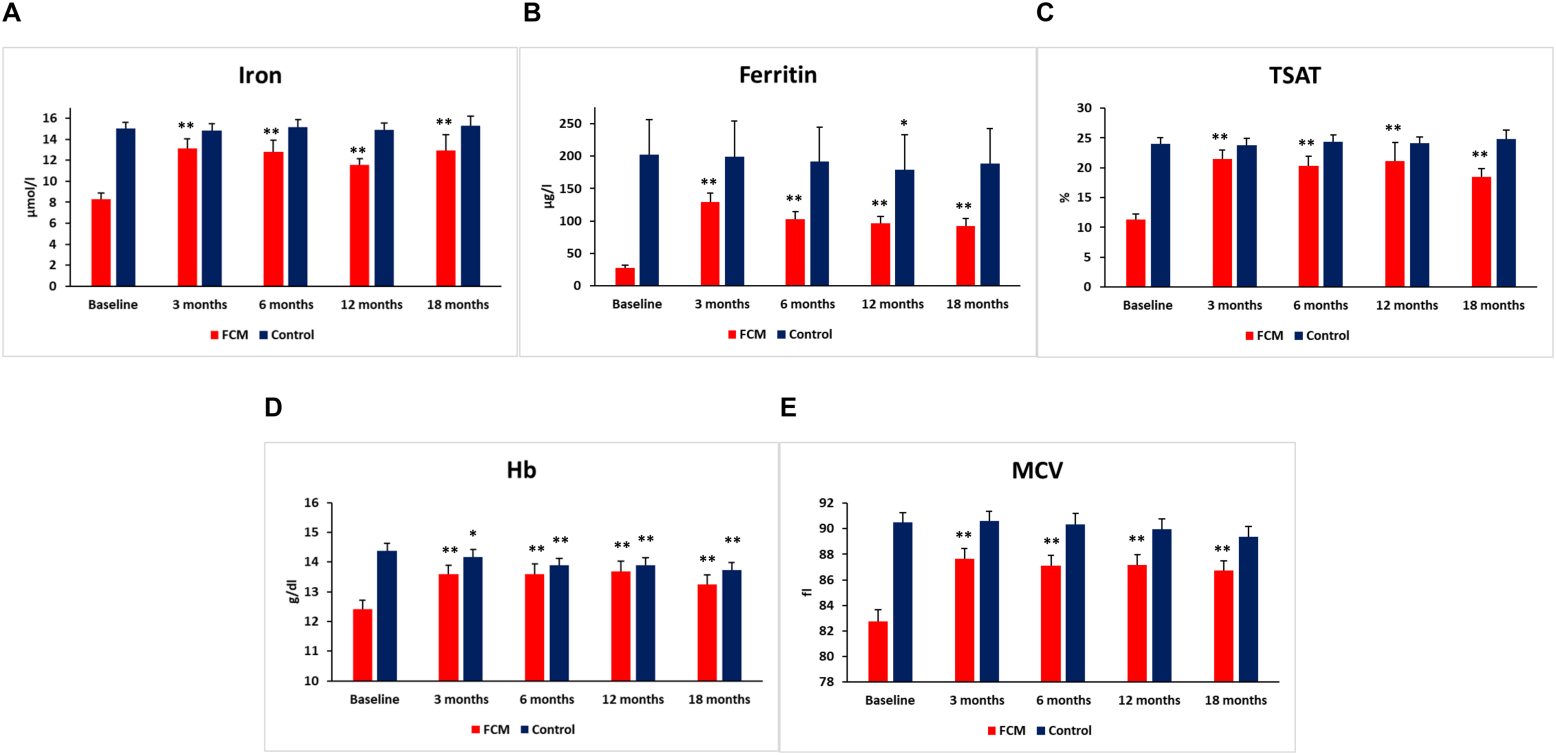
Impact of FCM on iron status and haematinics. Development of iron status (A: iron; B: ferritin; C: TSAT), haemoglobin concentrations (D), and MCV (E) from baseline to 18 month follow‐up in the FCM vs. control group. Numbers represent mean values ± SEM. *P < 0.05; **P > 0.01. FCM, ferric carboxymaltose; MCV, mean corpuscular volume; TSAT, transferrin saturation.

All PAH patients in the intervention group had ID at baseline, and 90% had a TSAT < 20%. No overt anaemia was present, although haemoglobin concentrations and MCV were slightly higher in the matched control group (both *P* < 0.001) (*Table* S2). Patients with ID underwent routine diagnostic work‐up for ID (*Table* S3). Upon i.v. iron supplementation with FCM (mean dose 924 ± 155 mg), all measures of iron status (iron, ferritin, and TSAT) as well as haemoglobin and MCV significantly improved in the intervention group and remained stable throughout the observation period (all *P* < 0.001 for interaction), whereas there were no significant changes in the control group (*Figure* 1A–1E and *Table* S3). No patient received another type of iron substitution. Peak values for ferritin and TSAT were reached at the 3 month follow‐up and showed a slightly declining trend afterwards. Fourteen patients in the intervention group received a second FCM infusion after an average time of 9.6 ± 4.8 months, of which four patients needed a maximum of three FCM infusions due to re‐occurrence of ID (mean total FCM dosage of each patient 1196 ± 563 mg; range 500–3000 mg). Nine out of 14 patients requiring repetitive FCM infusions were on oral anticoagulation (five phenprocoumon and four rivaroxaban). Patients in the control group showed only small changes in haematinics during the 18 month follow‐up.

### Exercise capacity and pulmonary arterial hypertension symptoms

**Figure 2:**
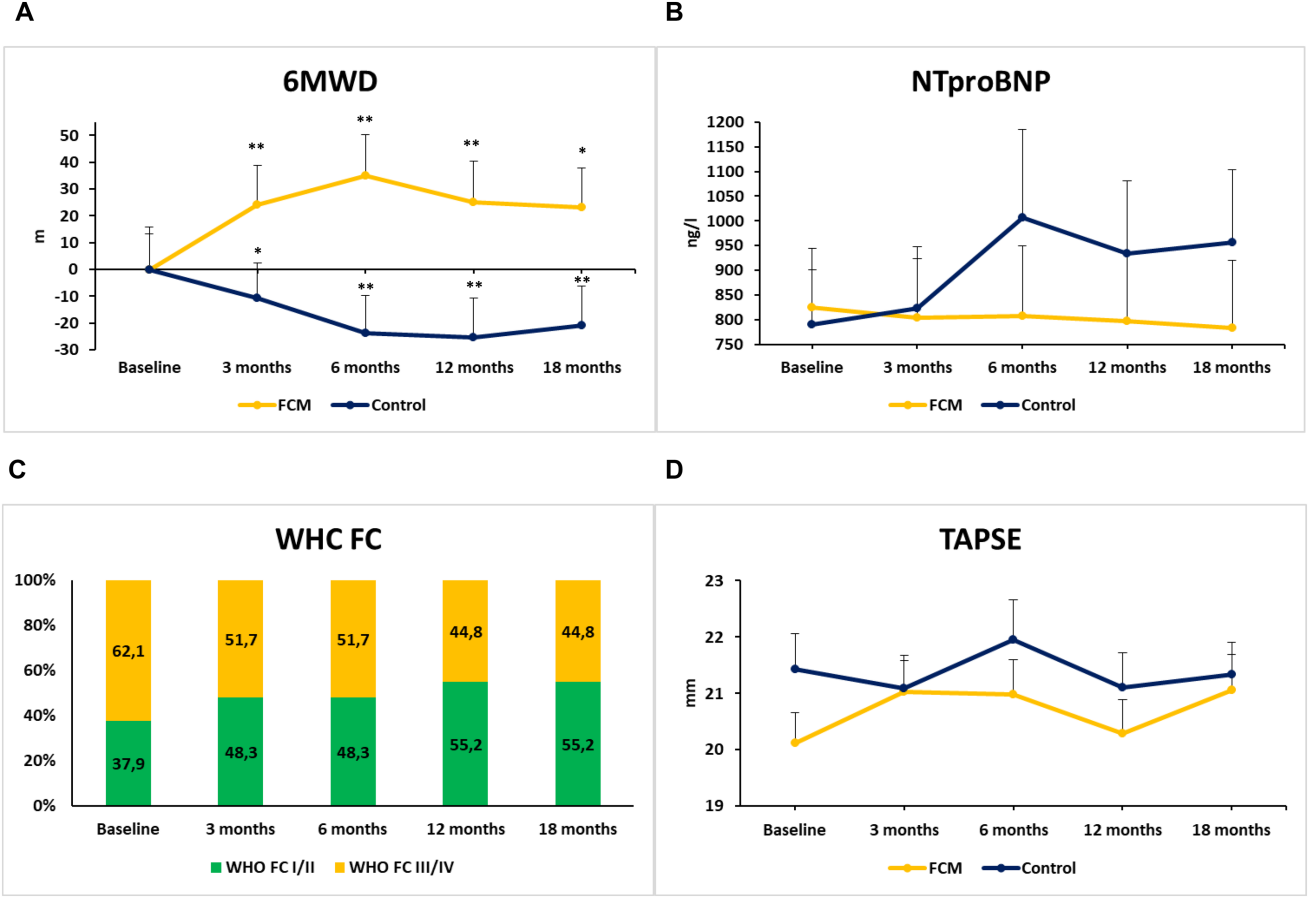
Impact of FCM on clinical, laboratory, and echocardiographic measures. Development of (A) 6 min walking distance (6MWD), (B) NTproBNP serum concentrations, (C) WHO functional class (WHO‐FC; in the FCM group), and (D) tricuspid annular plane systolic excursion (TAPSE) from baseline to 18 month follow‐up in the FCM vs. control group. Numbers represent mean values ± SEM. *P < 0.05; **P < 0.01 vs. baseline.

The restoration of iron status in the intervention group was associated with an improvement of exercise capacity and symptom burden within the first 6 months and throughout the observation period. While the mean baseline value of 6MWD was lower in iron‐deficient patients compared with the control group, during 18 months under FCM treatment the level of 6MWD in the intervention group at least reached the baseline level of the control group. At 6 and 18 months after FCM infusion, 6MWD improved from 378 ± 16 at baseline to 413 ± 15 and 401 ± 15 m, respectively (both *P* < 0.05), whereas the 6MWD in the control group slightly deteriorated (*Figure* 2A, *Table* S3).

Both groups displayed elevated NTproBNP concentrations at baseline. In the intervention group, there was a slight trend towards a reduction, while a 21% increase was observed in the control group at the conclusive follow‐up visit (*Figure* 2B, *Table* S3). As expected from previous observations, iron‐deficient PAH patients were more symptomatic at baseline. In patients treated with FCM, the proportion of patients in WHO‐FC I or II increased from 38% at baseline to 55% at 18 months (*Figure* 2C), whereas a slight deterioration (52% at baseline and 43% at 18 months) was found in the control group.

Pulmonary arterial systolic pressure was markedly elevated in both groups and slightly higher in the intervention group. Upon FCM treatment, no essential changes were observed with regards to echocardiographic measurements including pulmonary arterial systolic pressure, tricuspid annular plane systolic excursion, right atrium area, right ventricular end‐diastolic diameter, or left ventricular ejection fraction in both groups (*Figure* 2D, *Table* 3).

**Table 3:** Echocardiographic variables at baseline and during follow‐up

Variable	Baseline	3 months	6 months	12 months	18 months
Intervention (*n* = 58)					
RA area (cm^2^)	22.6 ± 1.1	23.4 ± 1.3	22.6 ± 1.1	21.6 ± 0.9	22.3 ± 1.1
RVEDD (mm)	41.1 ± 1.0	41.1 ± 0.9	42.3 ± 0.9	40.8 ± 0.9	41.8 ± 1.0
∆*P*_max_TV (mmHg)	57.1 ± 3.5	57.7 ± 3.6	55.0 ± 3.4	55.1 ± 3.8	55.9 ± 3.4
PASP (mmHg)	62.4 ± 3.7	62.3 ± 3.7	60.2 ± 3.6	60.8 ± 4.0	60.8 ± 3.6
TAPSE (mm)	20.1 ± 0.5	21.0 ± 0.5	21.0 ± 0.6	20.3 ± 0.6	21.1 ± 0.6
LVEF (%)	63.9 ± 1.1	63.4 ± 1.1	63.4 ± 1.1	63.6 ± 1.0	63.3 ± 1.0
Control (*n* = 59)					
RA area (cm^2^)	23.8 ± 1.1	23.1 ± 1.0	23.7 ± 1.1	23.1 ± 1.0	23.6 ± 0.9
RVEDD (mm)	41.6 ± 1.0	41.6 ± 1.2	40.7 ± 1.0	41.5 ± 1.0	40.8 ± 1.0
∆*P*_max_TV (mmHg)	55.2 ± 2.7	53.6 ± 2.6	52.8 ± 3.2	55.2 ± 3.3	54.4 ± 3.3
PASP (mmHg)	59.6 ± 2.9	57.8 ± 2.8	57.2 ± 3.3	60.4 ± 3.4	59.3 ± 3.5
TAPSE (mm)	21.4 ± 0.6	21.1 ± 0.6	22.0 ± 0.7	21.1 ± 0.6	21.3 ± 0.6
LVEF (%)	65.1 ± 1.2	66.8 ± 1.0	65.5 ± 1.1	65.6 ± 1.2	65.1 ± 1.1

∆*P*_max_TV, systolic tricuspid pressure gradient; LVEF, left ventricular ejection fraction; PASP, pulmonary artery systolic pressure; RA, right atrium; RVEDD, right ventricular end‐diastolic diameter; TAPSE, tricuspid annular plane systolic excursion.

We also monitored the potential impact of baseline RV function on the treatment effect of FCM and found that the improvement of the 6MWD in the intervention group was similar in patients with impaired or preserved RV function.

### Pulmonary arterial hypertension‐associated hospitalizations and pulmonary arterial hypertension risk status

**Figure 3:**
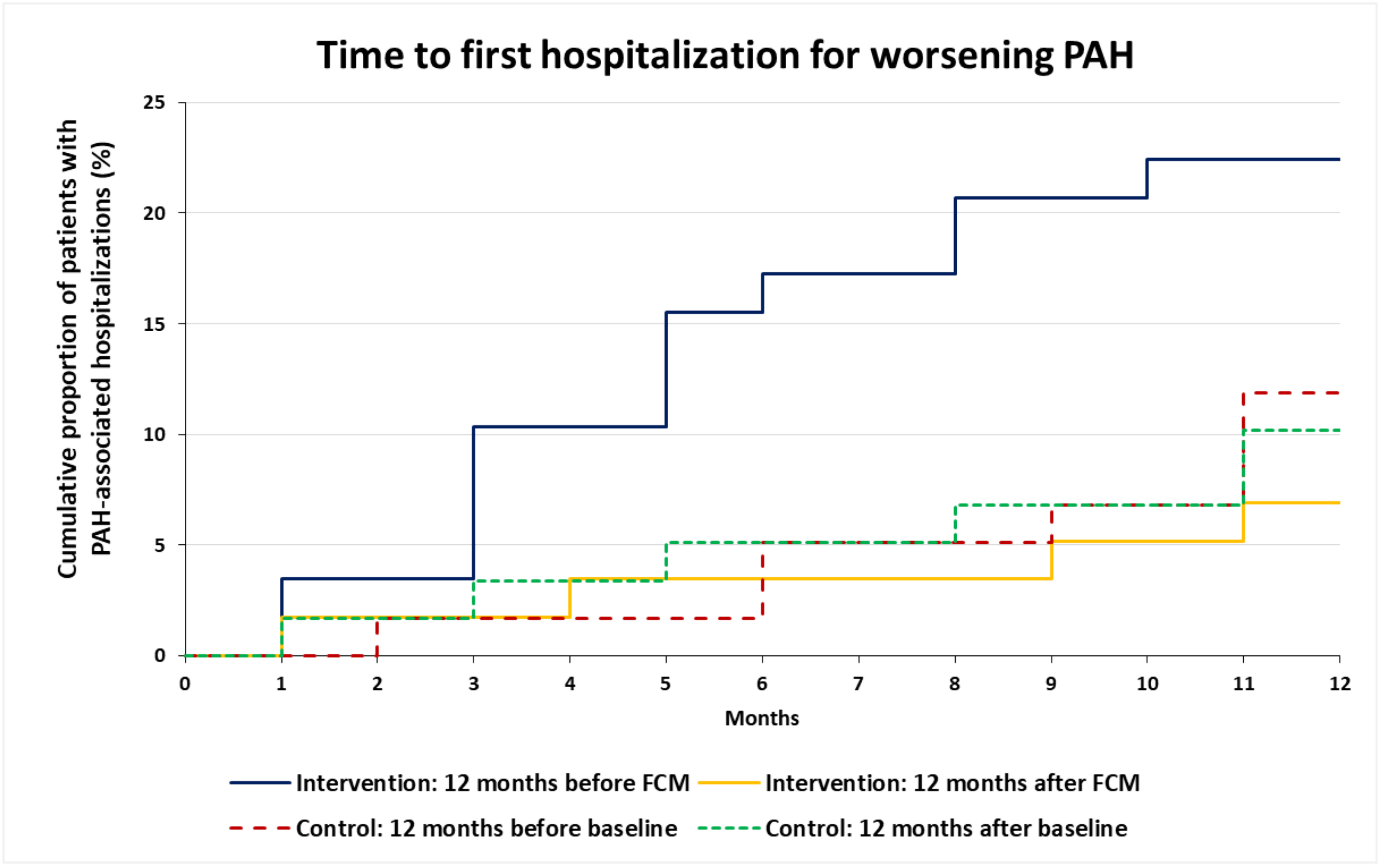
PAH‐associated hospitalizations during the 12 month period prior to vs. post‐FCM treatment (intervention group) or pre‐baseline vs. post‐baseline (control group). Wilcoxon signed‐rank test: *P* = 0.029 (intervention); *P* = 1.000 (control). FCM, ferric carboxymaltose; PAH, pulmonary arterial hypertension.

In order to evaluate whether the improvement in clinical measures is reflected in a reduction of morbidity events, we reviewed the frequency of hospitalizations for worsening PAH. *Figure* 3 illustrates the cumulative development of first hospitalizations for worsening PAH over time, depicting a lower hospitalization rate after restoration of iron status. In detail, 13 patients (22.4%) had at least one PAH‐associated hospitalization with a total of 16 PAH‐hospitalizations during the 12 month pre‐treatment phase presumably under conditions of ID vs. 4 patients (6.9%) with a total of 5 hospitalizations for worsening PAH during the 12‐months after FCM therapy. In the control group, seven PAH‐associated hospitalizations occurred in seven patients (11.9%) in the 12 months pre‐FCM, while six patients (10.2%) were hospitalized due to worsening PAH (nine hospitalizations) during the 12 months post‐FCM. Overall, there were 37 and 17 hospitalizations for any reason in the FCM and control groups prior to FCM vs. 27 and 17 hospitalizations during the 12 months after the first FCM administration, respectively. Non‐PAH hospitalizations were due to gastrointestinal bleeding, pneumonia, bone fracture, and cancer.

**Figure 4:**
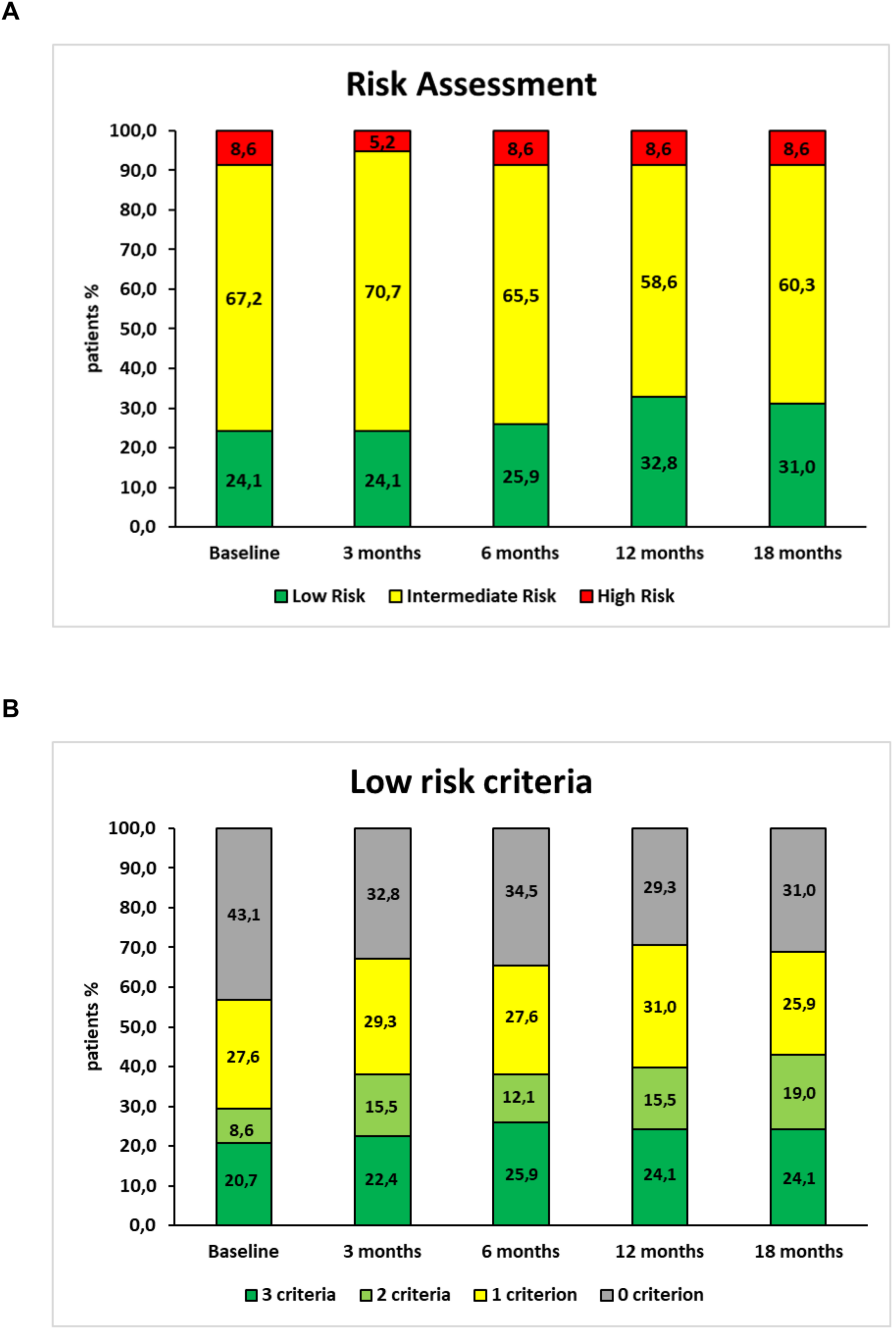
Risk stratification in the FCM group based on the development of clinical variables from baseline to 18 month follow‐up according to the ESC/ERS guidelines,^1^ (A) utilizing the SPAHR methodology of analysis.^27^ Each variable was graded from 1 to 3 where 1 = ‘Low risk’, 2 = ‘Intermediate risk’, and 3 = ‘High risk’. Dividing the sum of all grades by the number of available variables for each patient rendered a mean grade. The mean grade was rounded off to the nearest integer, which was used to define the patient's risk group^27^; (B) utilizing the FNPH methodology of analysis which focuses on low‐risk criteria only.^25^ Analysis based on the course of three non‐invasive clinical variables. Low risk defined by WHO‐FC I‐II; 6MWD > 440 m, NTproBNP < 300 ng/mL.

The ESC/ERS risk status—an important prognosticator in PAH—also improved. By using the SPAHR methodology, the proportion of patients in the FCM group with low‐risk status improved from 24.1% at baseline to 31.0% at 18 months (*Figure* 4A). Consistent with these results, the proportion of patients in the FCM group with two or three variables in the low‐risk category increased from 29.3% to 43.1% from baseline to 18 months after FCM treatment, while the proportion of patients with only one or no low‐risk criteria decreased from 70.7 to 56.9% (*Figure* 4B).

In the FCM group, one patient died during the 18 month follow‐up (14 months after baseline) while no patient died in the control group.

### Tolerability

Intravenous FCM was generally well tolerated. No serious adverse events or hypersensitivity reactions, and no cases of hypophosphatemia occurred. Minor complaints reported by two patients, which resolved shortly after i.v. application, included transient flu‐like symptoms in one patient, and a temporary, minor skin discoloration at the infusion site in one patient, caused by a small amount of the infusion running paravasally, which had been corrected by resiting the cannula. Hospitalizations in the intervention group were not related to FCM treatment in any case.

## Discussion

This study assessed the long‐term safety and potential efficacy of parenteral iron repletion with FCM in iron‐deficient PAH patients. Consistent with a former pilot study,^15^ our data suggest that a single infusion of FCM restores iron status and leads to a sustained improvement of clinically relevant variables, PAH risk status, and hospitalization rates. Only a limited number of patients required repetitive iron supplementations due to recurrent ID during an extended observation period.

As expected from previous studies,^8^, ^10^, ^15^ iron‐deficient PAH patients were more symptomatic, had lower exercise capacity and higher NTproBNP concentrations, which may reflect the additional impact of ID on disease burden. The lower body weight in ID patients may be explained by energetic insult to skeletal muscle and its association with cachexia.^13^ Correction of ID was associated with improvements of 6MWD and WHO‐FC, which beneficially affected PAH risk status, according to ESC/ERS guidelines.^1^, ^25^, ^26^, ^27^, ^28^ The magnitude of changes in the 6MWD upon correction of ID was similar to improvements reached by PAH‐targeted drugs in several randomized controlled trials^3^, ^4^, ^5^ and is equal or above the estimated minimal clinical important difference in the 6MWD for PAH patients.^23^ Of note, this potential long‐term effect of FCM was achieved on top of targeted therapy, and a ‘ceiling effect’ must be considered in patients who have already improved their exercise capacity.^29^ It is unlikely that the observed changes are due to escalation of PAH‐targeted drugs that may have been carried out in parallel, as they occurred rapidly after FCM administration, and treatment adjustments were rare and less frequent in the FCM group compared with the control group. FCM therapy was well tolerated and safe in PAH patients.

### Hospitalization rates and pulmonary arterial hypertension risk status

Consistent with studies in HF patients, our data suggest a reduction of PAH‐associated hospitalizations upon FCM treatment. These data have to be interpreted with caution, as iron status of the patients before FCM treatment is not known. The hospitalization rate before iron supplementation (22.4%) is somewhat higher when compared with the placebo groups in studies on iron replacement in HF,^17^, ^18^, ^20^ but similar to the hospitalization rates of prevalent patients in large PAH trials including GRIPHON and SERAPHIN.^3^, ^4^, ^5^ A landmark analysis of the latter studies revealed that morbidity events (and hospitalizations in particular) are highly predictive of mortality in PAH,^30^ supporting the clinical relevance of our findings.

Furthermore, the improvement of clinical measures upon FCM supplementation in our study had impact on patients' PAH risk status and led to a remarkable shift from intermediate‐risk to low‐risk, according to the risk assessment strategy proposed in the European PH guidelines.^1^ Because the risk status is known to predict mortality rates in PAH,^25^, ^26^, ^27^, ^28^ bringing the patient into the low‐risk category is considered as an important treatment goal in PAH.

Taken together, our data on clinical improvement in patients with PAH are consistent with previous studies in patients with chronic or acute HF.^31^, ^32^ Moreover, the reductions of HF hospitalizations upon correction of ID, confirmed in meta‐analyses,^18^, ^33^ are consistent with our preliminary data in PAH. Hence, numerous trials in patients with HF—and now PAH—suggest that treatment with FCM is associated with clinical improvement and reduction in the risk of hospitalizations for worsening left‐sided or right‐sided HF.

### Reason for iron deficiency and route of iron administration

In the absence of other reasons, in PAH patients, ID may be explained by impaired dietary iron absorption due to raised concentrations of hepcidin,^10^ a key regulator of iron homeostasis.^14^ The liberation of hepcidin is regulated by plasma and liver iron concentrations and involves signalling via bone morphogenetic protein (BMP). Interestingly, in IPAH BMP receptor type II (BMPR2) expression is reduced, and loss‐of‐function mutations in BMPR2 have been linked to >70% of heritable PAH and 10–20% of IPAH cases.^34^ BMP‐6 was recognized as the main BMP regulating hepatic hepcidin expression in vivo,^35^ and BMPR2 knockdown in HepG2 cells increased BMP‐6 stimulated hepcidin expression.^10^ Thus, raised concentrations of hepcidin may impair the bioavailability of oral iron in PAH.^8^

When correcting iron status, the route of administration may therefore be crucial. While the FAIR‐HF, CONFIRM‐HF, EFFECT‐HF, and FERRIC‐HF trials demonstrated that parenteral iron therapy with FCM effectively restored ferritin and TSAT levels, which was associated with short‐term and long‐term clinical improvements,^17^, ^18^, ^19^, ^20^ the IRONOUT‐HF trial showed that even high‐dose oral iron supplementation failed to improve ferritin and TSAT levels and exercise capacity.^36^ Smaller studies in PAH patients with ID also found that oral iron supplementation was inefficient to restore ferritin concentrations.^7^, ^8^, ^10^ Collectively, these data do not support the use of oral iron supplementation in HF or PAH patients with ID, although a small study recently suggested efficacy of oral ferric maltol in PH.^37^ In our study, a sufficient repletion of ferritin in a range of >100 μg/L was observed 3 months after FCM‐treatment and maintained until the end of the observation period. Nevertheless, the fact that both ferritin and TSAT values as well as haemoglobin and MCV remained lower than in the control group suggests that we may have dosed higher, potentially underestimating the therapeutic potential of iron repletion in PAH.

### Iron deficiency, mitochondrial, and muscle function

The mechanisms on how iron repletion improves the clinical status of patients with left‐sided or right‐sided HF remain speculative. Beneficial long‐term effects may be triggered by improved cardiac function, reducing right ventricular afterload and optimizing cellular oxygen supply in the right ventricle.^8^, ^11^, ^38^ ID was also shown to be associated with skeletal and inspiratory muscle weakness in chronic diseases including HF.^12^, ^13^ Potentially, the rise of exercise capacity in our study could be explained by increased haemoglobin and myoglobin, improved oxygen metabolism of the heart, skeletal and inspiratory muscles, or reduced pulmonary vascular resistance.^8^, ^10^, ^38^, ^39^, ^40^ In the present study, we excluded patients with overt anaemia (haemoglobin < 8.0 g/dL). Certainly, some PAH patients may be relatively anaemic because of the lack of reactively increased haemoglobin due to chronic hypoxia. However, studies in HF and PAH demonstrated clinical improvements irrespective of the presence of anaemia^15^, ^17^, ^18^, ^20^ indicating that iron substitution serves more than haemoglobin synthesis. In this context, the RED‐HF trial has demonstrated no improvements of clinical outcomes in HFrEF patients and mild‐to‐moderate anaemia after treatment with darbepoetin alfa^41^ suggesting that correcting ID, rather than anaemia, is of relevance in HF patients.

Iron plays a critical role in synthesis of haemoglobin in erythrocytes and myoglobin in the heart and skeletal muscles, which impact on oxygen delivery, utilization, and intracellular storage.^40^ Beyond that, iron is involved in functions of mitochondrial oxidative enzymes, the respiratory chain, oxidative phosphorylation, vascular homeostasis, nitric oxide generation, and the citric acid cycle.^38^, ^42^ ID is associated with reduced mitochondrial function in human cardiomyocytes,^11^ altered sarcomere structure,^42^ and impaired left ventricular systolic function.^36^, ^40^ In vitro, ID directly affects human cardiomyocyte function, impairing mitochondrial respiration and reducing contractility and relaxation. Restoration of intracellular iron can reverse these effects.^43^ Accordingly, in the presence of ID, oxygen supply and function of the high‐energy demanding skeletal and cardiac myocytes are also severely disturbed, resulting in diminished exercise capacity,^8^, ^38^ worsened symptoms and poor outcome also in PAH patients harbouring impaired RV function.^9^, ^10^ Although we did not detect significant changes in RV function at rest, subtle changes may be difficult to capture in RV, and effects on RV contractile reserve and RV‐PA coupling may be expected.

### Limitations

The main limitations of this study are its retrospective character, the lack of a placebo group, and the single‐centre approach. Moreover, we did not measure soluble transferrin receptor concentrations in iron‐deficient patients, which represents a more reliable marker for ID in the presence of inflammation,^44^ and the role of ferritin for the definition of ID is currently challenged.^45^ However, this should not have influenced our data because patients with markedly elevated CRP (>25 mg/l) were excluded, and the vast majority had TSAT < 20%. With regard to hospitalizations, data have to be interpreted with caution, as the number of events was low, and iron status is not known for the 12 month pre‐treatment period.

## Conclusions

Parenteral iron repletion with FCM in iron‐deficient PAH patients was well tolerated in addition to targeted PAH therapy, and may provide long‐term clinical benefit, including improved risk status and reduced PAH‐associated hospitalizations. Control of iron status may represent an effective supportive treatment strategy, in addition to targeted therapy. Our results provide proof‐of‐concept concerning the long‐term efficacy and safety of FCM in patients with PAH and ID and may inform further randomized controlled trials.

## Conflicts of interest

TK: Remunerations for lectures from Actelion. MW, KN, TV, MH, SB: Nothing to disclose. FG: Remunerations for lectures Actelion, Bayer, GSK, and United Therapeutics; grants to institution from Actelion, Bayer, Novartis und United Therapeutics. HtF: Remunerations for lectures from Actelion, Bayer. DD: Remunerations for lectures from Actelion. SR: Remunerations for lectures and/or consultancy from Abbott, Acceleron, Actelion, Arena, Bayer, BMS, GSK, Janssen, MSD, Novartis, Pfizer, United Therapeutics, and Vifor; grants to institution from Actelion, AstraZeneca, Bayer, Janssen, Novartis, and United Therapeutics.

## Supporting information


**Table S1.** Drug treatment at baseline in the intervention (FCM) and control group.

**Table S2.** Conditions detected during diagnostic work‐up for ID.

**Table S3.** Iron status and clinical measures at baseline and during follow‐up. Data are presented as median (interqurtile range), or means ± SEM; *p < 0.05; **p < 0.01 vs. baseline).

**Figure S1** Overview of screened and excluded PAH patients. Defined exclusion criteria were kidney dysfunction (serum creatinine > 2.0 mg/dl), considerable liver disease (serum glutamic oxaloacetic transaminase/glutamic pyruvic transaminase > 70 U/l), marked anemia (hemoglobin < 8.0mg/dl), marked inflammation (C‐reactive protein (CRP) > 25 mg/l), lost to follow‐up, or no stable PAH therapy for at least 3 months.
