# Supplementary material for: Ferric carboxymaltose in patients with pulmonary arterial hypertension and iron deficiency: a long‐term study
Source: J Cachexia Sarcopenia Muscle. 2021 Sep 9;12(6):1501–12. doi: 10.1002/jcsm.12764 (PMC8718050; doi:10.1002/jcsm.12764)
Supplement: Supplementary file 1 — Table S1. Drug treatment at baseline in the intervention (FCM) and control group. [file JCSM-12-1501-s004.docx]

**Supplementary Material**

**Table S1**. Drug treatment at baseline in the intervention (FCM) and control group.

EPO, erythropoietin; NSAID, nonsteroidal anti-inflammatory drugs; DAPT, dual antiplatelet therapy; ASA, acetylsalicylic acid, ACEi, angiotensin-converting enzyme (ACE) inhibitors; ARB, angiotensin receptor blocker; MRA, mineralocorticoid receptor antagonist; PPI, proton-pump inhibitor.

|  | **Intervention**  (n=58) | **Control**  (n=59) | **p-value** |
| --- | --- | --- | --- |
| **Drug Treatment:** | | | |
| EPO, n (%) | 0 (0.0%) | 0 (0.0%) | ≈1.000 |
| NSAID, n (%) | 3 (5.2%) | 0 (0.0%) | 0.119 |
| DAPT, n (%) | 1 (1.7%) | 2 (3.4%) | ≈1.000 |
| ASA, n (%) | 15 (25.9%) | 16 (27.1%) | ≈1.000 |
| Anticoagulation, n (%) | 30 (51.7%) | 30 (50.8%) | ≈1.000 |
| ACEi, n (%) | 8 (13.8%) | 17 (28.8%) | 0.070 |
| ARB, n (%) | 4 (6.9%) | 11 (18.6%) | 0.095 |
| Beta blocker, n (%) | 19 (32.8%) | 24 (40.7%) | 0.444 |
| Statin, n (%) | 15 (25.9%) | 17 (28.8%) | 0.836 |
| MRA, n (%) | 11 (19.0%) | 23 (39.0%) | 0.025 |
| Loop diuretic, n (%) | 36 (62.1%) | 42 (71.2%) | 0.331 |
| Thiazide diuretics, n (%) | 11 (19.0%) | 32 (54.2%) | 0.000 |
| Thyrostatic agents, n (%) | 2 (3.4%) | 0 (0.0%) | 0.244 |
| Thyroid hormones, n (%) | 13 (22.4%) | 17 (28.8%) | 0.526 |
| Cortisone, n (%) | 6 (10.3%) | 10 (16.9%) | 0.421 |
| PPI, n (%) | 23 (39.7%) | 21 (35.6%) | 0.705 |
| Allopurinol, n (%) | 3 (5.2%) | 13 (22.0%) | 0.013 |
| Metformin, n (%) | 2 (3.4%) | 2 (3.4%) | ≈1.000 |
| Insulin, n (%) | 3 (5.2%) | 4 (6.8%) | ≈1.000 |
