# Supplementary material for: Ferric carboxymaltose in patients with pulmonary arterial hypertension and iron deficiency: a long‐term study
Source: J Cachexia Sarcopenia Muscle. 2021 Sep 9;12(6):1501–12. doi: 10.1002/jcsm.12764 (PMC8718050; doi:10.1002/jcsm.12764)
Supplement: Supplementary file 2 — Table S2. Conditions detected during diagnostic work‐up for ID. [file JCSM-12-1501-s003.docx]

**Table S2**. Conditions detected during diagnostic work-up for ID.

|  | **Intervention group** | **Control group** |
| --- | --- | --- |
| Crohn's disease | 1 | 0 |
| Colon diverticulitis | 1 | 0 |
| Gastritis / ulcer | 2 | 4 |
| Reflux esophagitis | 2 | 2 |
| Esophageal varices | 1 | 0 |
| Angiodysplasia of the colon | 1 | 1 |
| Thrombocytopenia | 1 | 0 |
| Monoclonal gammopathy | 1 | 1 |
| Polycythemia vera | 1 | 1 |
| Chronic lymphatic leukemia (CLL) | 1 | 0 |
| Non-Hodgkin lymphoma | 0 | 1 |
