# Supplementary material for: Ferric carboxymaltose in patients with pulmonary arterial hypertension and iron deficiency: a long‐term study
Source: J Cachexia Sarcopenia Muscle. 2021 Sep 9;12(6):1501–12. doi: 10.1002/jcsm.12764 (PMC8718050; doi:10.1002/jcsm.12764)
Supplement: Supplementary file 3 — Table S3. Iron status and clinical measures at baseline and during follow‐up. Data are presented as median (interqurtile range), or means ± SEM; *p < 0.05; **p < 0.01 vs. baseline). [file JCSM-12-1501-s001.docx]

**Table S3**. Iron status and clinical measures at baseline and during follow-up. Data are presented as median (interqurtile range), or means ± SEM; *p<0.05; **p<0.01 vs. baseline).

|  | **Baseline** | **3 Months** | **6 Months** | **12 Months** | **18 Months** |
| --- | --- | --- | --- | --- | --- |
| **Iron status** | | | | | |
| **Intervention** (n=58) | | | | | |
| MCV, fl | 83.0  (80.0; 87.0) | 87.0  (84.0; 91.0)** | 88.0  (84.0; 91.0)** | 87.0  (84.0; 90.0)** | 86.0  (82.0; 90.0)** |
| Hemoglobin, g/dl | 12.2  (10.7; 13.8) | 13.5  (12.4; 14.5)** | 13.6  (11.9; 14.9)** | 13.8  (11.9; 14.8)** | 13.2  (11.5; 14.4)** |
| Iron, µmol/l | 7.5  (4.5; 9.4) | 12.0  (8.9; 16.3)** | 11.1  (7.8; 15.9)** | 11.8  (8.4; 14.5)** | 10.2  (7.4; 14.4)** |
| Ferritin, µg/l | 21  (14; 31) | 100  (44; 180)** | 76  (24; 155)** | 74  (38; 135)** | 61  (25; 136)** |
| TSAT, % | 10.0  (6.0; 13.0) | 21.0  (12.3; 28.0)** | 20.0  (12.0; 27.0)** | 18.5  (12.0; 23.8)** | 16.0  (11.3; 23.0)** |
| **Control** (n=59) | | | | | |
| MCV, fl | 91.0  (87.0; 93.5) | 91.0  (87.5; 94.0) | 91.0  (87.0; 94.0) | 89.0 (87.0; 93.5) | 89.0 (87.0; 92.5) |
| Hemoglobin, g/dl | 14.1  (13.2; 15.6) | 14.1  (12.8; 15.3)* | 13.9  (12.8; 14.8)** | 13.8  (12.4; 15.0)** | 13.6  (12.5; 15.0)** |
| Iron, µmol/l | 14.4  (11.9; 17.6) | 14.4  (12.0; 17.3) | 14.1  (11.1; 18.2) | 13.8  (11.5; 18.7) | 14.1  (10.5; 18.4) |
| Ferritin, µg/l | 100  (64; 202) | 92  (54; 166) | 94  (55; 187) | 80  (46; 175)* | 80  (44; 200) |
| TSAT, % | 23.0  (18.0; 28.0) | 23.0  (17.0; 29.0) | 23.0  (18.5; 31.0) | 23.0 (19.0;29.0) | 24.0  (17.0; 28.5) |
| **Clinical measures** | | | | | |
| **Intervention** (n=58) | | | | | |
| WHO-FC | 2.6 ± 0.1 | 2.6 ± 0.1 | 2.5 ± 0.1 | 2.5 ± 0.1* | 2.4 ± 0.1* |
| 6MWD, m | 378 ± 16 | 402 ± 15** | 413 ± 15** | 403 ± 15** | 401 ± 15* |
| NTproBNP, ng/l | 825 ± 120 | 803 ± 120 | 808 ± 141 | 797 ± 138 | 784 ± 137 |
| **Control** (n=59) | | | | | |
| WHO-FC | 2.6 ± 0.1 | 2.6 ± 0.1 | 2.6 ± 0.1 | 2.6 ± 0.1 | 2.6 ± 0.1 |
| 6MWD, m | 403 ± 13 | 393 ± 13* | 380 ± 14** | 378 ± 15** | 383 ± 15** |
| NTproBNP, ng/l | 790 ± 110 | 823 ± 124 | 1007 ± 178 | 934 ± 147 | 956 ± 147 |
