# Supplementary material for: Ferric carboxymaltose in patients with pulmonary arterial hypertension and iron deficiency: a long‐term study
Source: J Cachexia Sarcopenia Muscle. 2021 Sep 9;12(6):1501–12. doi: 10.1002/jcsm.12764 (PMC8718050; doi:10.1002/jcsm.12764)
Supplement: Supplementary file 4 — Figure S1 Overview of screened and excluded PAH patients. Defined exclusion criteria were kidney dysfunction (serum creatinine > 2.0 mg/dl), considerable liver disease (serum glutamic oxaloacetic transaminase/glutamic pyruvic transaminase > 70 U/l), marked anemia (hemoglobin < 8.0mg/dl), marked inflammation (C‐reactive protein (CRP) > 25 mg/l), lost to follow‐up, or no stable PAH therapy for at least 3 months. [file JCSM-12-1501-s002.docx]

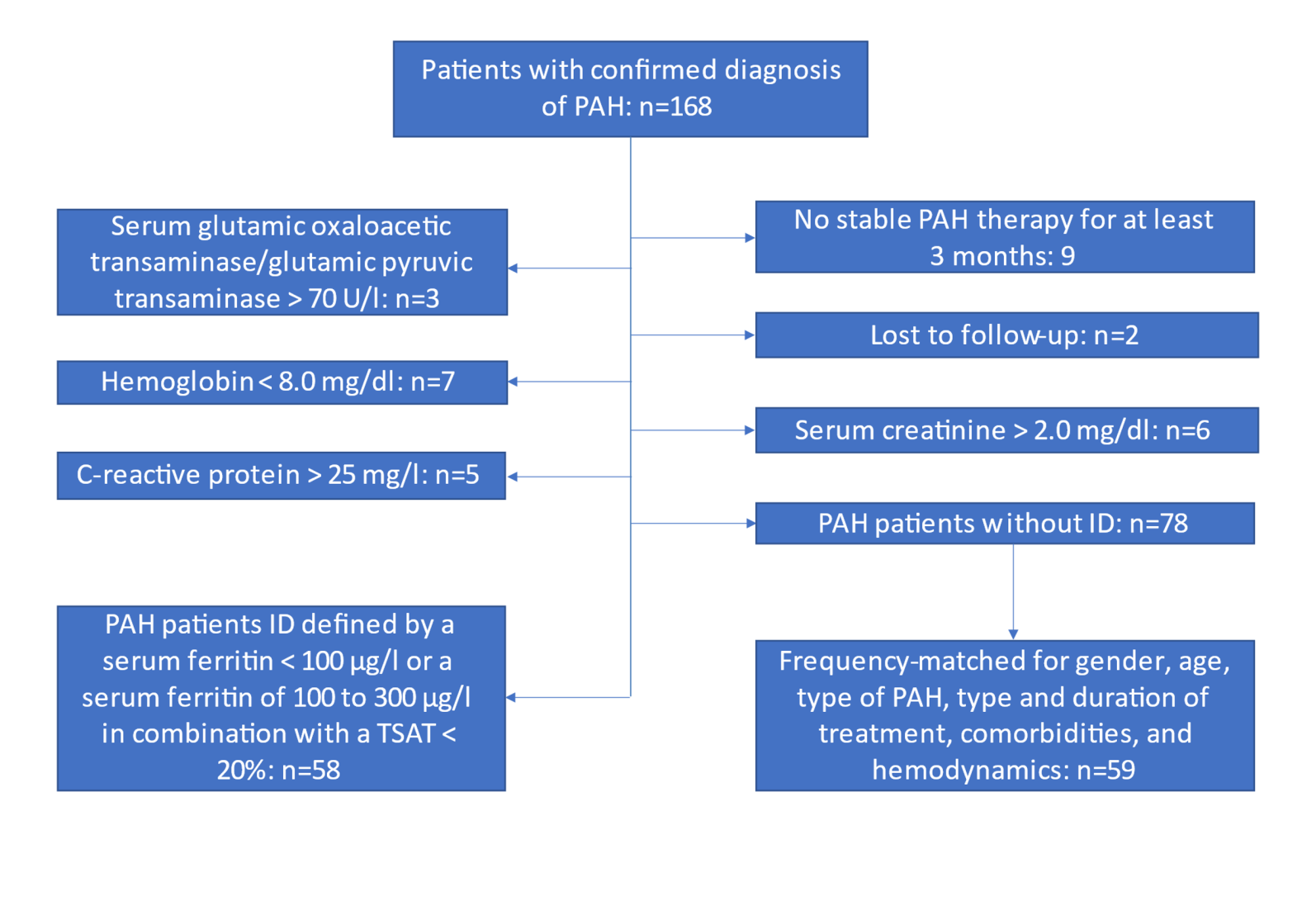


**Figure S1** Overview of screened and excluded PAH patients. Defined exclusion criteria were kidney dysfunction (serum creatinine > 2.0 mg/dl), considerable liver disease (serum glutamic oxaloacetic transaminase/glutamic pyruvic transaminase > 70 U/l), marked anemia (hemoglobin < 8.0 mg/dl), marked inflammation (C-reactive protein (CRP) > 25 mg/l), lost to follow-up, or no stable PAH therapy for at least 3 months.
